# Deciphering the Chemotherapeutic Mechanism of Ferulic Acid: Insight Into the Role Against Multiple Human Cancers

**DOI:** 10.1002/cai2.70056

**Published:** 2026-04-20

**Authors:** Sanzida Khatun, Md Sohel, Zitu Barman, Umme Salma, Lubatul Arbia, Md. Rifat Sarker, Jasmin Akter Jame, Badhan Rani Dey, Sultana Parvin, Md. Shah Poran Shuvo, Md. Shahidul Islam, Tania Mannan, Snygdha Rani Das, Md. Mahmudul Hasan

**Affiliations:** ^1^ Department of Biochemistry and Molecular Biology Primeasia University Dhaka Bangladesh; ^2^ Department of Biochemistry and Molecular Biology Dhaka International University, Satarkul Dhaka Bangladesh; ^3^ Department of Biochemistry and Molecular Biology Mawlana Bhashani Science and Technology University Dhaka Bangladesh

**Keywords:** cancer, ferulic acid, nanoformulation, synergistic effect

## Abstract

Extensive research continues to address the challenges of developing standard cancer drugs. However, until more effective standard drugs are developed, ferulic acid (FA) may be a potential option for controlling the symptoms of cancer patients. According to our review, FA is available in natural sources and has flexible structures that possess diverse pharmacological activities. FA is effective against 16 different cancer types and has been validated in cell culture, preclinical, and clinical models. Chemotherapeutics activities of FA are regulated through varieties of mechanisms, including targeting signaling pathways, such as AKT/PI3K/mTOR/ERK/STAT NF‐κB; apoptosis, such as FAS/FASL, TRADD, Bcl2, Bax, Caspases, and PARP; metastasis, such as MMPs(1,2,9), Wnt/‐β catenin, angiogenesis (E&N Cadherin, vimentin, Snail, and Slug), cell proliferation (cyclin D1, E1, and CDKs(2,4,6)), inflammatory molecules (TNF‐α, NF‐κB,1α, IL‐10, IL‐8, and IL‐6), regulating tumor suppressor genes (p‐RB, p21, and p53), autophagy (LC3‐II, p62, Beclin1, and Atg12–Atg5), glycolysis (lncRNA 495810 and PKM2), heat shock protein (Hsp60, Hsp70, and Hsp90), and some nonspecific pathways, such as oncogene suppression and antioxidant efficacies. Nanoformulation of FA increased its solubility, stability, and bioavailability, thereby enabling controlled release and making FA more effective against cancer. Additionally, FA exerted synergistic effects with other natural compounds, vitamins, radiotherapy, and chemotherapies, and reversed resistance to existing chemotherapies via diverse mechanisms, including targeting multidrug resistance proteins, apoptosis, reactive oxygen species production, hypoxia, microRNA, the β‐catenin pathway, oncogene activation, and sensitizing chemotherapies and radiotherapies. Given that FA has validated the experimental model and demonstrated preliminary efficacy, these findings suggest a possible supportive role for phytochemicals pending the development of fully effective pharmaceutical therapies.

## Graphical abstract

Ferulic acid (FA), a naturally occurring compound, exhibits anticancer activity against 16 cancer types through multiple molecular pathways. Its enhanced delivery via nanoformulations and synergistic effects with other therapies suggest FA as a promising, food‐based adjunct for cancer symptom control until fully approved pharmaceutical options are available. This image was created in BioRender, M. Sohel https://BioRender.com/8prxhnf.

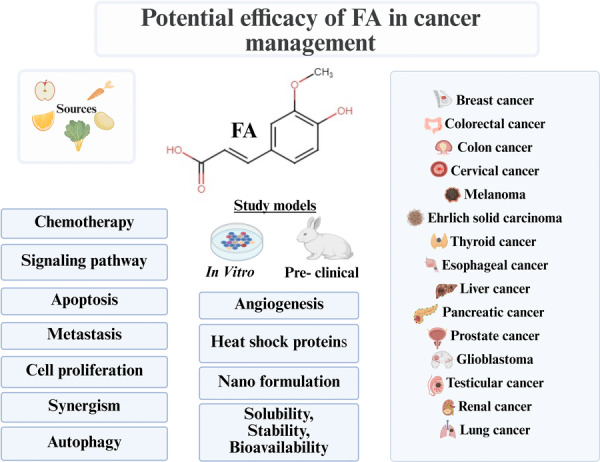

**Abbreviations**
4NQO: 4‐nitroquinoline‐1‐oxide
Akt: protein kinase B
ALT: alanine aminotransferase
AST: aspartate aminotransferase
BALB/c: mouse strain
Bcl‐2: B‐cell lymphoma 2
CDK: cyclin‐dependent kinases
CYCS: Cytochrome CDLD‐1/human colorectal adenocarcinoma cellline
EMT: epithelial–mesenchymal transition
FA: ferulic acid
FACL: fas ligand
FAS: fas cell surface death receptor
IGF‐II: insulin‐like growth factor II
IUPAC: International Union of Pure and Applied Chemistry
MAPK: mitogen‐activated protein kinase
MIA PaCa‐2: human pancreatic carcinoma cell line
MMP: matrix metalloproteinases
NF‐κB: nuclear factor kappa‐light‐chain‐enhancer of activated B cells
ODC: ornithine decarboxylase
PI3K: phosphoinositide 3‐kinase
RGD: arginine‐glycine‐aspartic acid (motif)
ROS: reactive oxygen species
SAR: structure–activity relationship
STAT3: signal transducer and activator of transcription 3
TG2: transglutaminase 2
TXA2: thromboxane A2
VEGF: vascular endothelial growth factor

## 1 Introduction

Cancer has become one of the most pressing global health obstacles, with its incidence steadily increasing over the years [1]. According to 2024 cancer statistics, there were around 611,720 cancer‐related fatalities and 2 million newly diagnosed cancers in the United States alone, where the global number is uncountable [2]. This significant milestone of incidence, as well as death due to lack of approved drugs and side effects of all existing treatments, emphasizes the urgent need for effective preventive and therapeutic care to combat this growing health crisis [3]. From the beginning to now, numerous chemotherapeutic drugs have been developed and employed in tumor treatment. However, no definitive or complete cures are currently available in clinical practice. The cost of all emerging therapeutic strategies can be offset by selecting natural compounds derived from plants that have attracted considerable interest for their potential contributions to cancer prevention and treatment [4, 5, 6]. These bioactive phytochemicals, often characterized by their minimal toxicity and diverse pharmacological properties, offer a complementary approach to conventional treatments [7]. Additionally, recent comprehensive analyses such as the review “Cancer Treatments: Past, Present, and Future” by Sonkin et al. [8] from the U.S. National Cancer Institute provide a historical perspective on how natural compounds have progressively gained recognition in oncology. This work underscores the transition from purely synthetic chemotherapeutics to the integration of plant‐derived bioactives as complementary or synergistic agents in modern cancer treatment. Ferulic acid (FA) can be a potential lead compound because of its safety and possible therapeutic application.

FA is also known as *trans*‐FA or 4‐hydroxy‐3‐methoxycinnamic acid. This phytoestrogen is widely found in plant‐based sources, such as wheat, oats, rice bran, oranges, corn, apples, seeds, nuts, vegetables (spinach, beetroot), and herbs, like Angelica and Ginkgo biloba [33]. Previously, we published several articles on chemotherapeutic activities against human malignancies with specific phytoestrogens, for example, quercetin [34], hesperetin [35], sesamin [36], calycosin [37], phloretin [38], baicalein [39], esculetin [40], biochanin A [5], and other groups of phytoestrogens [6, 41, 42]. We plan to build a library of phytochemicals with strong chemotherapeutic activities. Therefore, currently, focusing on FA is due to its unique pharmacological characteristics. One of them, its favorable structure, comprises an unsaturated side chain, *trans*‐cinnamic acid with methoxy and hydroxy substituents at positions 3 and 4, respectively, on the phenyl ring, and both *cis* and *trans* isomeric forms [43, 44, 45]. Due to its structural diversity, FA has a multifunctional mechanistic profile, including antioxidant and redox‐balancing capacity, anti‐inflammatory action, and chemosensitizing potential. In line with cancer, FA exerts significant chemotherapeutic effects across a variety of malignancies, including breast [9], colorectal [14], colon [12], cervical [15], melanoma [19], ehrlich solid carcinoma (ESC) [46], osteosarcoma [21], thyroid [22], lung [47], esophageal [26], liver [27], pancreatic [28], prostate [29], testicular [31], and renal cancer [32]. These effects are evident in FA's ability to modulate critical molecular pathways involved in tumor initiation, progression, and development. FA has moved from preclinical promise to early‐stage clinical and nutraceutical applications, appearing in formulations for adjunct cancer therapy and preventive oncology. Furthermore, FA can align with modern therapeutics trends, as it bridges nutrition, pharmacology, and clinical oncology, aligning with the “food‐as‐medicine” and supporting conventional treatment strategies. Furthermore, earlier reviews of the literature unveiled possible mechanisms of chemotherapeutic activities. This review aims to systematically explore the present understanding of the anticancer mechanisms of FA, according to in silico, in vitro, preclinical, and clinical models, and its potential applications in modern cancer biology. On the basis of these studies, we determine how FA can be delivered to improve efficacy, synergistically enhancing its therapeutic use and reversing the resistance mechanisms of existing chemotherapies and radiotherapies in cancer treatment. By delving into its molecular mechanism, this article explained why people need FA as a food supplement for cancer treatment.

## 2 Overview of Ferulic Acid

### 2.1 Sources of FA

Common foods that contain FA include coffee, fruits, whole grains, and some vegetables [48]. Cereals and other grains such as rice, wheat bran, corn, and oats are rich in FA. Fruits are also a good source of FA, including apples, oranges, pineapples, and berries. Daily‐use vegetables such as spinach, broccoli, and tomatoes are also natural sources of FA. Seeds like sesame, flax, and sunflower are high sources of FA. Beverages, including tea, coffee, and certain alcoholic drinks, such as wine, are additional sources of FA due to the presence of plant polyphenols.

### 2.2 Structure and Physicochemical Properties

**Figure 1:**
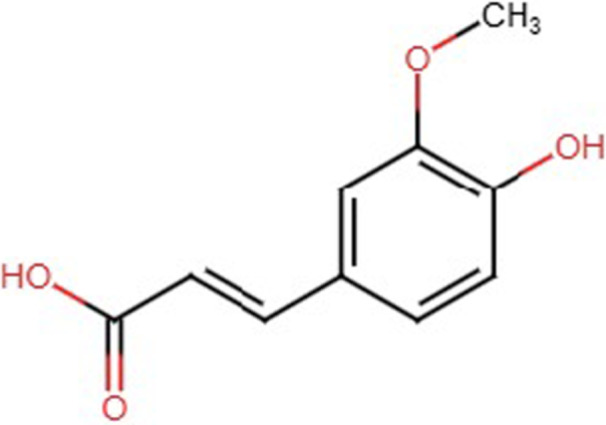
Chemical structure of ferulic acid.

The International Union of Pure and Applied Chemistry name of FA is (*E*)‐3‐(4‐hydroxy‐3‐methoxyphenyl) prop‐2‐enoic acid, with the chemical formula C_10_H_10_O_4_. The chemical structure of this compound resembles a benzene ring containing a hydroxyl group, a methoxy group, and a carboxylic acid side chain, connected by a conjugated double bond. FA is readily soluble in organic solvents, such as ethanol, chloroform, and hexane. The chemical structure of FA is shown in Figure 1.

### 2.3 Structure–Activity Relationship of FA

**Figure 2:**
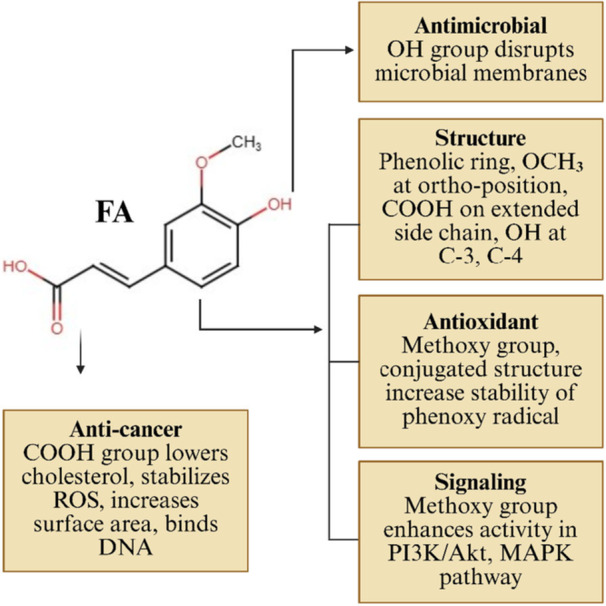
Structure–activity relationship of FA and its biological functions. The diagram illustrates how FA's structural components—phenolic ring, methoxy, hydroxyl, and carboxylic acid groups—contribute to its antioxidant, antimicrobial, anticancer, and signaling activities. through stabilization of free radicals, membrane disruption, DNA interaction, and modulation of signaling pathways. Akt, protein kinase B; FA, ferulic acid; MAPK, mitogen‐activated protein kinase; PI3K, phosphoinositide 3‐kinase; ROS, reactive oxygen species. Created in BioRender. Sohel, M. (2026) https://BioRender.com/8prxhnf.

On the basis of its chemical structure, FA has several functional groups that confer biological activities, such as antioxidant, anti‐inflammatory, and anticancer. FA's structure includes a phenolic ring, a methoxy group positioned ortho to it, a carboxylic acid group on an extended side chain, and two hydroxyl groups at the C‐3 and C‐4 positions. The antioxidant capacity of FA is associated with the electron‐donating methoxy group, which stabilizes the phenoxy radical and is further enhanced by electron‐donating substituents [49]. The conjugated double bond of FA stabilizes free radicals through resonance effects, thereby enhancing its antioxidant properties. The methoxy group of FA is connected to oncogenic signaling pathways, such as PI3K/Akt and mitogen‐activated protein kinase (MAPK), which regulate cell cycle and mediate apoptosis [20]. Another functional group is the hydroxyl group, which interacts with phospholipids in the cell membrane, thereby disrupting it [50]. The carboxylic group of FA facilitates the formation of esters that reduce free cholesterol in tissue and inhibit lipid peroxidation, acting as an anchor. Both the carboxylic and hydroxy groups of FA interact with DNA molecules and halt cancer progression [51]. FA has a tropological polar surface area of around 66.8 Å^2^, which is associated with its anticancer activity, as it inhibits telomerase enzymes [52]. A summary of the link between FA's structure and activity, as well as its biological roles, is depicted in Figure 2.

### 2.4 The Modification of FAs on the Survival and Apoptosis Pathway

The targeted modulation of critical survival and apoptotic signaling pathways largely accounts for the anticancer effects of FAs. Evidence reveals that several cancer models support FA's ability to block pro‐survival signals and activate programmed cell death. FA often suppresses the MAPK and PI3K/AKT signaling pathways, which are vital for cell survival and proliferation. FA significantly elevated reactive oxygen species (ROS) levels and induced apoptosis in estrogen‐negative breast cancer cells (MDA‐MB‐231), primarily via the MAPK/STAT3/NF‐κB pathways, while simultaneously reducing phosphorylated protein kinase B (p‐AKT) [10]. Likewise, FA induced apoptosis in colorectal (CT26) and colon cancer models by phosphorylating ERK and JNK, two components of the MAPK pathways, which, in turn, regulated the ratio of pro‐ and antiapoptotic proteins [12]. By directly modulating essential apoptotic machinery, such as the B‐cell lymphoma 2 (Bcl‐2) family and executioner caspases, FA consistently triggers apoptosis. Research has demonstrated that FA upregulates proapoptotic Bax and downregulates antiapoptotic Bcl‐2 and Mcl‐1 in a variety of cancer models, including osteosarcoma (143B and MG63) and pancreatic (MIA PaCa‐2) models [20, 28]. This change in the Bax/Bcl‐2 ratio increases mitochondrial outer membrane permeability, thereby activating the caspase cascade. FA has been shown to activate executioner caspase‐3 in colorectal (HT‐29 and HCT‐116) and thyroid tumor (TT) cancer cells [14, 22]. In addition, FA can activate other important tumor suppressors and apoptotic regulators. In pancreatic and prostate cancer cells, FA therapy restored p53 and PTEN activity, hence increasing apoptotic signaling [28, 29]. A comprehensive analysis of medullary TT cells revealed FA‐mediated overexpression of p53, poly (ADP‐ribose) polymerase (PARP), and the BH3‐only proteins PUMA and NoXA, which resulted in caspase‐3/9 activation [22]. This integrated evidence across various cancers emphasizes FA's significance as a potent multitarget drug that induces apoptosis via numerous interrelated pathways.

### 2.5 FA in Redox Regulation and ROS‐Mediated Cytotoxicity

FA serves a dual function in cellular redox control, functioning as both an antioxidant and a prooxidant, with its actions dramatically influenced by context and concentration. Although it can neutralize free radicals to protect healthy cells, FA can also trigger a fatal increase in ROS, leading to oxidative stress and cellular apoptosis. A key cause of FA's cytotoxicity is the impairment of the mitochondrial electron transport chain, which leads to the production of reactive ROS. FA significantly increased intracellular ROS levels in MD A‐MB‐231 breast cancer cells, leading to cell death via the MAPK/STAT3/NF‐κB pathway [10]. Similarly, FA increased apoptosis and DNA damage in cervical cancer cells (Henrietta Lacks [HeLa] and Caski), in part via activating ROS42. This ROS‐dependent apoptosis is not exclusive to carcinomas; it has been demonstrated that FA causes pyroptosis, a specific type of cell death, in lung cancer cells via activation of the JNK/Bax mitochondrial pathway [47]. FA's ability to cause ferroptosis, a novel type of iron‐dependent cell death marked by lipid peroxidation, further demonstrates its prooxidant potential. By causing ferroptosis, FA treatment decreased the viability and migration of esophageal squamous cell carcinoma (EC‐1 and TE‐4) cells. In addition to the suppression of antioxidant defenses, such as decreased superoxide dismutase (SOD) activity and glutathione (GSH) levels, this was demonstrated by elevated levels of malondialdehyde (MDA), ROS generation, and intracellular iron accumulation [26]. Additionally, FA forms a feed‐forward loop that intensifies its toxic effects by influencing redox‐sensitive signaling pathways. Stress kinases, such as JNK, can be activated by ROS produced by FA. This can lead to mitochondrial dysfunction and increased ROS production, ultimately causing the cell to undergo apoptosis. This concerned effect in lung, esophagus, breast, and cervical cancer models highlights FA's potential as a powerful oxidative stress inducer in cancerous cells.

### 2.6 FA in Cell Cycle Control and Autophagy

FA halts cell division by targeting the cell cycle and triggers cell death by activating autophagy. FA induced cell cycle arrest at almost all phases, but this effect depended on cancer type and cell type. FA treatment in MDA‐MB‐231 cells arrests the cell cycle at G2/M by suppressing the p‐AKT pathway and other cell cycle‐related proteins [10] and S‐phase [52]. Similarly, in colorectal cancer, FA arrests the cell cycle at S and G2/M phases [14]. Arresting G0/G1 seems to be a common pathway for FA‐mediated cell cycle arrest; FA likely inhibited the proliferation of cervical (HeLa), lung (H1299), and prostate (PC‐3) cancer cells by arresting them in the G0/G1 phase [15, 23, 29]. This arrest is often mediated by downregulation of cyclins (e.g., Cyclin D1/E) and cyclin‐dependent kinases (CDK2, CDK4, and CDK6), and by upregulation of CDK inhibitors, like p21 and p27. Regarding autophagy, FA‐derived compounds called TBT‐F stimulate autophagy by upregulating LC3‐II and p62 [13]. In hepatocellular carcinoma (HCC), the HepG2 cell line, FA treatment induces autophagy by activating beclin‐1 and promoting the LC3‐I/LC3‐II conversion [27]. In cervical cancer, FA regulates autophagy by controlling markers, such as LC3‐II, Beclin1, and the Atg12–Atg5 complex [15].

### 2.7 FA in Antimetastasis and Anti‐Angiogenesis

FA also exhibits potent antimetastatic and anti‐angiogenic properties, which are essential for suppressing tumor invasion, dissemination, and vascularization.

#### 2.7.1 Inhibition of Metastasis

FA suppresses epithelial–mesenchymal transition (EMT), a major driver of metastatic progression. In MDA‐MB‐231 breast cancer cells, FA reduced key EMT markers—N‐cadherin, vimentin, Snail, and Slug—through MAPK pathway modulation, thereby limiting invasive potential [10]. Similar anti‐invasive effects were reported in cervical cancer models (HeLa and Caski), where FA inhibited MMP‐9 messenger RNA (mRNA) expression and reduced cellular invasion by inducing G0/G1 arrest and downregulating Cyclin D1/E [15]. These studies demonstrate FA's consistent ability to restrict metastatic phenotypes across multiple malignancies.

#### 2.7.2 Anti‐Angiogenic Activity

FA also interferes with tumor‐induced angiogenesis. In melanoma models, FA modulated ultraviolet (UV)‐induced signaling and reduced matrix metalloproteinases (MMP) like MMP‐1 and MMP‐9 expressions, both of which facilitate extracellular matrix remodeling required for vascular expansion [17]. Additionally, in lung cancer cells, FA‐mediated suppression of surviving and MMP‐2/9 indirectly disrupts angiogenic pathways by limiting endothelial survival signals and matrix degradation [23].

#### 2.7.3 Integrated Antimetastatic Potential

Collectively, these findings reveal that FA not only targets tumor cells directly but also disrupts the microenvironmental processes necessary for metastasis and angiogenesis. By inhibiting EMT, MMP activity, and angiogenic remodeling, FA emerges as a comprehensive antiprogression agent with broad therapeutic potential.

### 2.8 Additional Mechanisms: FA in Melanogenesis and Immunomodulation

**Figure 3:**
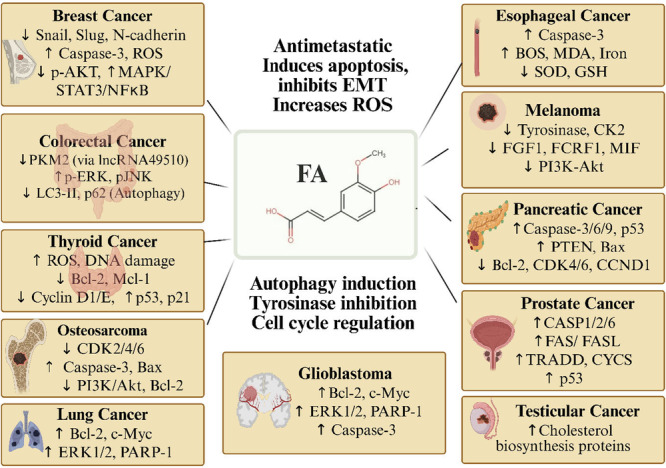
In vitro chemotherapeutic activity of ferulic acid (FA). Mechanistic overview of FA actions against various cancers, highlighting its roles in apoptosis induction, EMT inhibition, ROS generation, autophagy, tyrosinase inhibition, and cell cycle regulation across multiple cancer types, including breast, colorectal, thyroid, prostate, and lung cancers. Akt, protein kinase B; Bcl‐2, B‐cell lymphoma 2; CASP, Caspase; CCND1, cyclin D1; CDK, cyclin‐dependent kinase; c‐Myc, cellular myelocytomatosis oncogene; EMT, epithelial–mesenchymal transition; ERK1/2, extracellular signal‐regulated kinases 1 and 2; FA, ferulic acid; FGF1, fibroblast growth factor 1; GSH, reduced glutathione; LC3‐II, microtubule‐associated protein 1A/1B‐light chain 3‐II; MAPK, mitogen‐activated protein kinase; Mcl‐1, myeloid cell leukemia‐1; MDA, malondialdehyde; NF‐κB, nuclear factor kappa‐B; p‐AKT, phosphorylated protein kinase B; PARP, poly (ADP‐ribose) polymerase; p‐ERK, phosphorylated ERK; PI3K, phosphoinositide 3‐kinase; p‐JNK, phosphorylated c‐Jun N‐terminal kinase; PKM2, pyruvate kinase M2; PTEN, phosphatase and tensin homolog; BAX, Bcl‐2 associated X protein; ROS, reactive oxygen species; SOD, superoxide dismutase; STAT3, signal transducer and activator of transcription 3; TRADD, tumor necrosis factor receptor type 1‐associated DEATH domain. Created in BioRender. Sohel, M. (2026) https://BioRender.com/8prxhnf.

**Figure 4:**
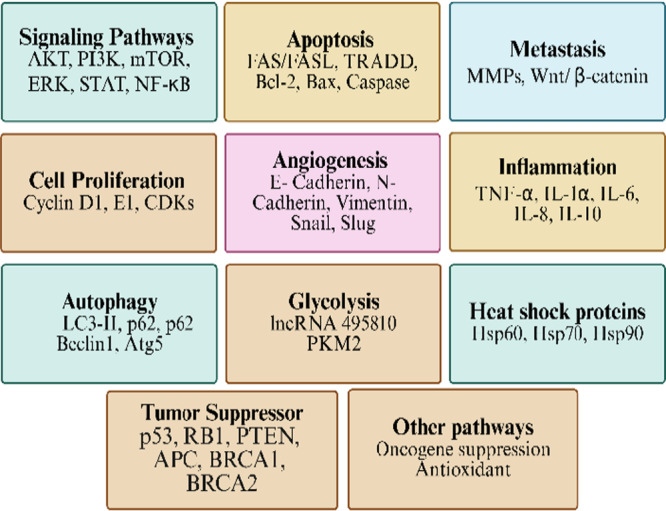
Molecular target of ferulic acid (FA) in cancer. Summary of molecular targets and pathways modulated by FA in cancer therapy, including signaling, apoptosis, metastasis, angiogenesis, inflammation, glycolysis, autophagy, tumor suppression, and heat shock protein regulation. Akt, protein kinase B; APC, adenomatous polyposis coli; BAX, Bcl‐2 associated X protein; Bcl‐2, B‐cell lymphoma 2; BRCA, breast cancer; CDK, cyclin‐dependent kinase; ERK, extracellular signal‐regulated kinase; Hsp60, heat shock protein 60; IL, interleukin; LC3‐II, microtubule‐associated protein 1A/1B‐light chain 3‐II; MMP, matrix metalloproteinase; mTOR, mammalian target of rapamycin; NF‐κB, nuclear factor kappa‐B; PI3K, phosphoinositide 3‐kinase; PKM2, pyruvate kinase M2; PTEN, phosphatase and tensin homolog; BAX, Bcl‐2 associated X protein; STAT, signal transducer and activator of transcription; TNF‐α, tumor necrosis factor‐alpha; TRADD, tumor necrosis factor receptor type 1‐associated DEATH domain. Created in BioRender. Sohel, M. (2026) https://BioRender.com/8prxhnf.

Despite these mechanisms, FA has specific mechanisms that vary by cancer type. For example, in melanoma, FA inhibits the tyrosine kinase enzyme and reduces the expression of microphthalmia‐associated transcription factor, thereby reducing melanin production [16, 17]. In addition, FA has an immunomodulatory effect that attenuates inflammatory response in immune cells by reducing LPS/IFN‐γ‐induced inducible nitric oxide synthase, cyclooxygenase‐2, and tumor necrosis factor‐α [19], suggesting a potential role in modulating the tumor microenvironment (Figures 3 and 4). The overall mechanisms of FA against human malignancies were summarized at Table 1.

**Table 1:** In vitro chemotherapeutic activities of ferulic acid on specific cancer.

Cancer type	Dose (µM)	Cell line	Molecular mechanism	Molecular target	Reference
Breast cancer	12.5–50	(MDA‐MB‐231)	↑Antimetastatic ↓Cell proliferation	↑Caspase‐3 ↓EMT ↓E‐cadherin ↓Snail ↓Slug	[9]
	12.5–50	MDA‐MB‐231	↑Apoptosis ↓Cell cycle G2/M arrest MAPK/STAT3/NF‐κB pathway	↑ROS ↓N‐cadherin ↓Vimentin ↓SNAI1 ↓p‐AKT	[10]
Colorectal cancer	NA	HT‐29, HCT116, SW620	↓Aerobic glycolysis	lncRNA 495810/PKM2 axis	[11]
	NA	CT26	↑Apoptosis ↓Cell Proliferation	↑p‐ERK ↑p‐JNK ↑BAX ↑BCL‐2	[12]
	NA	HCT116, HT‐29, Caco‐2	G2/M phase arrest ↑Autophagy	↑LC3‐II ↑p62	[13]
	IC50 = 350	HT‐29, HCT‐116	S and G2/M phase arrest ↑Apoptosis	↑Caspase‐3 ↑Caspase‐8 ↑Caspase‐9	[14]
Colon cancer	NA	CT26	↑Apoptosis ↓Cell growth	↑p‐ERK ↑p‐JNK ↓BCL‐2 ↑BAX	[12]
	NA	HeLa	↓MMP‐9 mRNA expression G0/G1 phase arrest ↓Cell invasion	↓Cyclin D1 ↓Cyclin E ↑p53 ↑p21 ↓LC3‐II ↓Beclin1 ↓Atg12–Atg5 complex	[15]
Melanoma	25–50	B16	↓Melanin production ↓Tyrosinase activity	↓Tyrosinase phosphorylation ↓CK2	[16]
	5–20	B16F10	↓Melanin synthesis ↓Tyrosinase expression ↓MITF expression	↓MMP‐1 ↓MMP‐9 ↑Procollagen ↑Hyaluronic acid ↑TIMP	[17]
	NA	A375, CHL‐1, SK‐MEL‐2	↓Cell proliferation	↓FGF1 ↓FGFR1 ↓PI3K‐Akt signaling	[18]
	100	RAW264.7		↓iNOS ↓Cox‐2 ↓TNF‐α ↓I‐kappa B degradation	[19]
Osteosarcoma	NA	143B, MG63	G0/G1 checkpoint arrest ↓Cell proliferation ↑Apoptosis	↓CDK2 ↓CDK4 ↓CDK6 ↑Caspase‐3 ↓PI3K/Akt pathway ↓Bcl‐2	[20]
	0–40	SaOS‐2, MG63	↓Cell viability ↑Apoptosis	↑Caspase‐3 ↑Bax ↓Bcl‐2	[21]
Thyroid cancer	150	*NA*	↓Cell invasion ↓Cell migration G0/G1 arrest ↑Apoptosis	↓URG4/URGCP ↓CCND1 ↓CDK4 ↓CDK6 ↓BCL2 ↓MMP‐2 ↓MMP‐9 ↑p53 ↑PARP ↑PUMA ↑NOXA ↑BAX ↑BID ↑CASP3 ↑CASP9 ↑TIMP1	[22]
Lung cancer	600	H1299	G0/G1 phase arrest ↓G2/M phase cells ↑Apoptosis	↑Bax ↓Survivin ↓MMP‐2 ↓MMP‐9	[23]
	NA	In vitro	↑Pyroptosis ↓Cell proliferation Morphological changes	↑GSDMD ↑ROS/JNK/Bax mitochondrial apoptosis pathway	[24]
	NA	A549	↑ERK/p38 signaling pathway	↑c‐JNK ↑Bax/Bcl‐2 ratio	[25]
Esophageal cancer	20–60	EC‐1, TE‐4	↓Cell viability ↓Cell migration ↑Apoptosis ↑Ferroptosis	↑Caspase‐3 ↑MDA ↑ROS ↑Iron accumulation ↓SOD ↓GSH	[26]
Liver cancer	≈ 206–824	HepG2	↓Cell proliferation ↑Apoptosis ↑Autophagy	↑Beclin‐1 ↑LC3‐I/LC3‐II ↑PINK‐1 ↑Parkin	[27]
Pancreatic cancer	150–1000	MIA PaCa‐2	↑Apoptosis ↑Cell cycle arrest	↑Bax ↑Caspase‐3, 8, 9, 10 ↑PTEN ↑p53 ↓Bcl‐2 ↓CCND1 ↓CDK4/6	[28]
Prostate cancer	300–500	PC‐3, LNCaP	↓Cell proliferation ↑Apoptosis ↑Cell cycle arrest	↑p53 ↓CDK2, CDK4, CDK6 ↑CASP1, CASP2, CASP8, CYCS, FAS, FASL, TRADD ↓BCL2, XIAP	[29]
Glioblastoma		Human glioblastoma cells	↓Cell viability ↑Apoptosis ↑Mitochondrial disruption	↓c‐Myc ↓Bcl‐2 ↑Caspase‐3 ↑PARP‐1 cleavage ↑ROS production ↑ERK1/2	[30]
Testicular cancer	10	MA‐10 Leydig cells	↑Gene Expression ↑Lipid and hormone production	↑Cholesterol and steroid biosynthesis genes/proteins ↑cAMP‐dependent star promoter activation	[31]
Renal cancer		A‐498	↓Cell proliferation ↑Apoptosis	↑Cleaved caspase‐3 ↓β‐catenin ↓c‐Myc	[32]

Abbreviations: AKT, protein kinase B; BAX, Bcl‐2 associated X protein; Bcl‐2, B‐cell lymphoma 2; BID, BH3‐interacting domain death agonist; cAMP, cyclic adenosine monophosphate; Caski, carcinoma of the skin; CASP3, Caspase‐3; CCND1, cyclin D1; CDK2, cyclin‐dependent kinase; c‐Myc, cellular myelocytomatosis oncogene; COX‐2, cyclooxygenase‐2; CYCS, cytochrome CDLD‐1/human colorectal adenocarcinoma cellline; EMT, epithelial–mesenchymal transition; ERK1/2, extracellular signal‐regulated kinases 1 and 2; FACL, fas ligand; FAS, fas cell surface death receptor; FGF1, fibroblast growth factor 1; FGFR1, fibroblast growth factor receptor 1; GSDMD, Gasdermin D; GSH, reduced glutathione; HeLa, Henrietta Lacks; iNOS, inducible nitric oxide synthase; JNK, c‐Jun N‐terminal kinase; LC3‐II, microtubule‐associated protein 1A/1B‐light chain 3‐II; lncRNA, long noncoding RNA; MAPK, mitogen‐activated protein kinase; MB, membrane bound; MCL‐1, myeloid cell leukemia‐1; MDA, malondialdehyde; MIA PaCa‐2, human pancreatic carcinoma cell line; MITF, microphthalmia‐associated transcription factor; MMP, matrix metalloproteinase; mRNA, messenger RNA; NA, not available; NF‐κB, nuclear factor kappa‐B; p‐AKT, phosphorylated protein kinase B; PARP, poly (ADP‐ribose) polymerase; p‐ERK, phosphorylated ERK; PI3K, phosphoinositide 3‐kinase; PINK1, PTEN‐induced kinase 1; p‐JNK, phosphorylated c‐Jun N‐terminal kinase; PKM2, pyruvate kinase M2; PTEN, phosphatase and tensin homolog; PUMA, p53 upregulated modulator of apoptosis; ROS, reactive oxygen species; SNAI1/Snail, snail family transcriptional repressor 1; SOD, superoxide dismutase; STAT3, signal transducer and activator of transcription 3; TIMP, tissue inhibitor of metalloproteinases; TNF‐α, tumor necrosis factor‐alpha; TRADD, tumor necrosis factor receptor type 1‐associated DEATH domain; URG4, upregulated gene‐4; URGCP, upregulator of cell proliferation XIAP, X‐linked inhibitor of apoptosis protein.

## 3 Preclinical and Clinical Evidence of Chemotherapeutic Activity for FA

**Figure 5:**
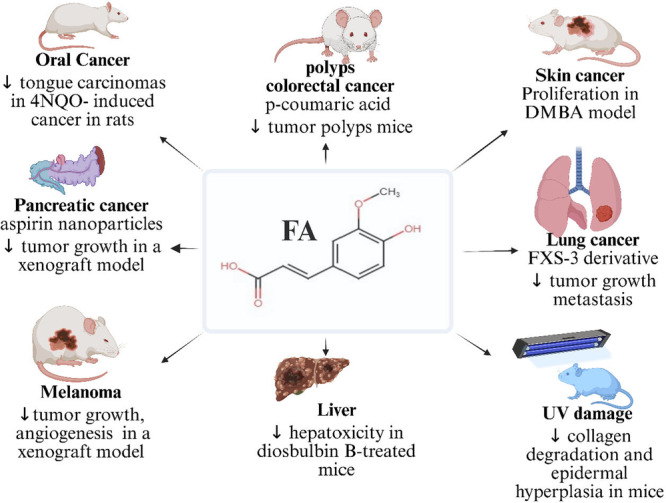
In vivo chemotherapeutic efficacy of FA: FA shows significant anticancer activity in oral, colorectal, skin, pancreatic, liver, melanoma, lung, and UV‐induced damage models. It reduces tumor growth, cell proliferation, angiogenesis, oxidative stress, and metastasis, and exhibits synergistic effects when combined with nanoparticles or derivatives. 4NQO4‐nitroquinoline‐1‐oxide; FA, ferulic acid; UV, ultraviolet. Created in BioRender. Sohel, M. (2026) https://BioRender.com/8prxhnf

Preclinical and clinical studies of FA are crucial for understanding its anticancer potential within complex biological systems. These studies allow researchers to evaluate their effectiveness, bioavailability, and safety in living organisms, providing insights beyond in vitro experiments. Preclinical and clinical evidence for FA's anticancer use remains minimal and largely indirect. However, we have some evidence showing that FA has chemotherapeutic activity in animals and humans. Mori et al. [53] conducted a pivotal late 20th‐century study on FA as a chemopreventive agent in male rats with 4‐nitroquinoline‐1‐oxide‐induced oral carcinogenesis. Their results demonstrated that dietary supplementation with 500 ppm FA significantly reduced the incidence of tongue carcinomas and severe dysplastic lesions compared with carcinogen‐exposed controls, highlighting FA's potential to inhibit both malignant transformation and preneoplastic progression in oral epithelial tissues. These findings provided foundational evidence for FA's dose‐dependent protective effects against chemical carcinogens in the oral cavity. Cui et al. [11] summarized that FA with *p*‐coumaric acid is effective in Male C57 BL/6 J mice. When this combination (10–60 mg/kg) is administered intragastrically, FA could decrease the tumor polyp count. By blocking long noncoding RNA (lncRNA) 495810 and the rate‐limiting enzyme M2 type pyruvate kinase, the natural product modifies aerobic glycolysis, according to a possible molecular mechanism. Preclinical studies demonstrate FA's broad‐spectrum anticancer efficacy across multiple models. Topical FA (10 μmol) inhibited 12‐*O*‐tetradecanoylphorbol‐13‐acetate–induced tumorigenesis in CD‐1 mice, reducing epidermal ODC activity and DNA synthesis by 42% [54]. For pancreatic cancer (PANC), Thakkar et al. [55] showed that oral FA (75 mg/kg) combined with aspirin nanoparticles reduced MIA PaCa‐2 xenograft growth by 45% in murine models. Additionally, FA exhibited photoprotective effects in ultraviolet B (UVB)‐irradiated skin, mitigating collagen degradation, abnormal elastin accumulation, and MMP‐2/9 overexpression while preventing epidermal hyperplasia [56]. These studies collectively highlight FA's multimodal mechanisms—spanning antiproliferative, antioxidant, and matrix‐protective pathways—across epithelial and visceral malignancies. FA and its derivatives demonstrate significant antitumor efficacy across diverse in vivo models. In melanoma xenografts, intragastric FA administration suppressed tumor growth by inhibiting FGF1‐mediated angiogenesis [18]. In colorectal cancer, FA‐loaded lipid nanocapsules (100 mg/kg) attenuated tumor progression in rats by downregulating cyclin D1, insulin‐like growth factor II (IGF‐II), and vascular endothelial growth factor (VEGF), while restoring the BAX/Bcl‐2 balance and reducing CD34+ microvessel density [57]. Wang et al. [58] conducted a study evaluating the hepatoprotective effects, which were evident in S180 cell‐transplanted mice: FA (8 mg/kg) mitigated diosbulbin B‐induced hepatotoxicity while enhancing tumor suppression. FA derivatives exhibited broad‐spectrum activity: FA15 (2‐methyl‐1‐butyl ferulic acid) reduced oxidative stress and papilloma formation in skin carcinogenesis models [19]. In colon cancer, FA modulated autophagy‐related genes and attenuated inflammation in BALB/c mice, concomitantly normalizing ALT/AST levels [14]. These findings were corroborated in triple‐negative breast cancer, where FA reduced MDA‐MB‐231 xenograft volume and weight via proapoptotic mechanisms [9]. In the context of cancer, clinical trials are essential for assessing new therapies, including natural compounds, providing hope for improved treatment effectiveness and fewer side effects. Murray et al. [59] used a topical preparation containing 0.5% FA (CEFer), 1% alpha‐tocopherol, and 15% l‐ascorbic acid to protect human skin against significant solar‐simulated UV radiation. Their study showed that FA could regulate erythema and sunburn cell formation, reduce thymine dimer mutations, and influence the tumor suppressor P53. Beyond its direct antitumor effects, FA exhibits potent anti‐inflammatory activity by suppressing key cytokines, including IL‐1α, IL‐6, IL‐8, IL‐10, and TNF‐α. This property synergizes with its dermatoprotective potential, as demonstrated in clinical studies of UV‐exposed human skin. A stabilized topical formulation combining FA (15%) with vitamins C (15%) and E (1%) significantly reduced photodamage biomarkers—lowering erythema by 52% (*p* < 0.01), sunburn cell counts by 74%, and thymine dimer formation by 63% compared with untreated controls. Further clinical evidence from Oresajo et al. [60] revealed that a phloretin‐enhanced FA/vitamin C antioxidant blend: (1) Attenuated UV‐induced MMP‐9 overexpression (↓82%) and p53 accumulation (↓67%). (2) Preserved epidermal immune function via CD1a+ Langerhans cell maintenance (↑3.2‐fold) and (3) showed synergistic protection against oxidative DNA damage. These findings position FA as both a chemopreventive agent and a cornerstone of photoprotective cosmeceuticals, with clinical efficacy against actinic carcinogenesis. The in vivo chemotherapeutic activity of FA is presented in Table 2 and Figure 5.

**Table 2:** In vivo chemotherapeutic activity of ferulic acid.

Cancer type	Dose	Study animal	Molecular mechanism	Molecular target	Reference
Oral carcinogenesis	500 ppm	NA	↓Tongue carcinomas ↓Preneoplastic lesions (severe dysplasia)	—	[53]
Colorectal cancer	10–60 mg/kg	C57BL/6J mice	↓Tumor polyps' count ↓Aerobic glycolysis	↓lncRNA 495810 ↓M2‐type pyruvate kinase	[11]
Skin carcinogenesis	10 µM (topical)	Female CD‐1 mice	↓Epidermal DNA synthesis ↓Tumor formation	↓TPA‐induced epidermal ornithine decarboxylase activity	[54]
Pancreatic cancer	75 mg/kg FA + 25 mg/kg aspirin nanoparticles	Pancreatic tumor xenograft, MIA PaCa‐2	↓Tumor growth (45% reduction)	—	[55]
Skin cancer		UVB‐irradiated mouse skin	↓Collagen fiber degradation ↓Elastic fiber accumulation ↓Epidermal hyperplasia	↓MMP‐2 ↓MMP‐9	[56]
Melanoma		Melanoma xenograft model	↓Tumor volume ↓Tumor weight ↓Angiogenesis	↓FGF1	[18]
Colorectal cancer	100 mg/kg	Rats	↓Metastasis ↑Apoptosis	↓Cyclin D1 ↓IGF‐II ↓VEGF ↑BAX/Bcl‐2 ratio ↓CD34	[57]
Liver cancer	8 mg/kg	ICR male mice, S180 cells	↓Tumor growth inhibition ↓Tumor weight	↓Diosbulbin B	[58]
Skin cancer		ICR mouse skin	↓Edema formation ↓Papilloma development	↓Free radicals (H_2_O_2_)	[19]
Colon cancer		BALB/c mice	↑Autophagy genes ↓Inflammatory response elements	Regulate ALT and AST	[12]
Breast cancer	12.5–50 µM	MDA‐MB‐231 xenograft model	↓Tumor volume ↓Tumor weight ↑Apoptosis	↑Caspase‐3 ↓EMT ↓E‐cadherin ↓Snail ↓Slug	[9]

Abbreviations: ALT, alanine aminotransferase; AST, aspartate aminotransferase; BALB/c, mouse strain; BAX, Bcl‐2 associated X protein; Bcl‐2, B‐cell lymphoma 2; CD34, cluster of differentiation 34; EMT, epithelial–mesenchymal transition; FA, ferulic acid; FGF1, fibroblast growth factor 1; ICR, Institute of Cancer Research; IGF, insulin‐like growth factor; lncRNA, long noncoding RNA; MB, membrane bound; MDA, malondialdehyde; MIA PaCa‐2, human pancreatic carcinoma cell line; MMP, matrix metalloproteinase; TPA, 12‐*O*‐tetradecanoylphorbol‐13‐acetate; UVB, ultraviolet B; VEGF, vascular endothelial growth factor.

## 4 Applications of FA in Cancer Treatment

### 4.1 Nano‐Based Application of FA in Cancer Treatment

Nano‐based FA has shown significant promise in cancer treatment due to its improved solubility, controlled release, enhanced bioavailability, and enhanced cellular uptake, ensuring prolonged therapeutic effects and making it a powerful tool in combating various cancers while advancing the potential of natural compounds in oncology. Rezaei et al. [61] reported that encapsulating FA (250 ppm) boosted its solubility by up to 15 times compared with its pure form, effectively reducing the viability of Michigan Cancer Foundation‐7 (MCF‐7) and 4T1 breast cancer cells while promoting apoptosis. Recent advances in nanoformulation have significantly enhanced FA's therapeutic potential against cancer. Vashisth et al. [62] developed polyethylene oxide‐based nanofibers for FA encapsulation, demonstrating sustained drug release that markedly increased apoptosis in MCF‐7 breast cancer cells compared with free FA. El‐Gogary et al. [57] further validated this approach using FA‐loaded lipid nanocarriers (100 mg/kg), which suppressed cyclin D1, IGF‐II, and VEGF expression in HCT116 and Caco‐2 cells while restoring BAX/Bcl‐2 balance.

In vivo, studies revealed that nanocarriers' improved antioxidant and anti‐inflammatory efficacy. According to Sweed et al. [63], co‐encapsulation of FA with solid lipid nanoparticles (SLNs) (25 µg/mL) may increase the cytotoxic effect on HT‐29 cancer, driven by multiple molecular processes, including apoptosis via regulation of cytochrome c release and caspase 9, and modulating specific genes, including P53 expression, more effectively than free FA or SLNs. FA combined with layered double hydroxide to form nanohybrid formulations significantly improved anticancer activity in cervical adenocarcinoma models and showed a dose‐dependent response across the 1–50 µM range. Most notably, these nanocomposites suppressed HeLa cell proliferation twice as effectively as free FA, highlighting the clear advantages of the hybrid design [64]. According to Ezhuthupurakkal, Preedia Babu et al. [65], zinc oxide nanoparticles bound to FA (ZnONPs‐FA) showed effective anti‐HCC effects through two key mechanisms: (1) ROS‐mediated apoptosis and (2) promotion of DNA damage in Huh‐7 and HepG2 cell lines. Importantly, this nanoformulation effectively suppresses diethyl nitrosamine (DEN)‐induced HCC progression, revealing its potential as a chemopreventive agent against liver carcinogenesis. To enhance the chemotherapeutic efficacy of FA, Dell'Albani et al. [66] employed FA‐loaded nanostructured lipid carriers (NLCs) (36 µM) in U87‐MG glioblastoma cells. Their findings revealed that these nanoparticles regulate TG2 localization and expression, thereby inducing apoptosis by modulating PARP‐1 and caspase‐3 cleavage.

**Figure 6:**
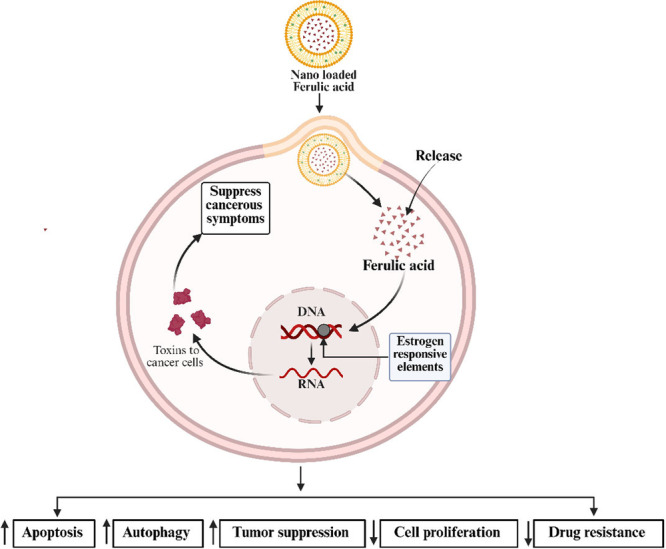
Nano‐based application of ferulic acid (FA) in cancer study. Graphical presentation of nanoloaded FA delivery into cancer cells, showing intracellular release, interaction with estrogen‐responsive elements, suppression of cancer symptoms, and downstream effects on apoptosis, autophagy, tumor suppression, cell proliferation, and drug resistance. Created in BioRender. Sohel, M. (2026) https://BioRender.com/8prxhnf.

Recent advances in nanoformulations have significantly enhanced FA's anticancer efficacy. Grasso et al. [30] demonstrated that while free FA merely downregulated cellular myelocytomatosis oncogene (c‐Myc) and Bcl‐2 expression in glioblastoma cells, FA‐loaded NLCs exhibited superior proapoptotic effects through caspase‐3 activation, PARP‐1 cleavage, and concurrent suppression of Bcl‐2, ERK1/2, and c‐Myc—effects validated through both molecular analyses and delayed luminescence techniques. Similarly, Thakkar et al. [55] showed that FA–aspirin coloaded solid lipid nanoparticles (c‐SLNs) induced apoptosis in PANC models via activation of the p‐RB/p21/ERK1/2 pathway, while enhanced cellular uptake enabled dose reduction. These nanoparticles effectively suppressed proliferation markers (proliferating cell nuclear antigen [PCNA] and marker of proliferation Ki‐67 [MKI67]) in both MIA PaCa‐2 and Panc‐1 cells and in SCID mouse xenografts. Further innovations include Zheng et al.'s [67] pH‐sensitive PFA&DOX nanoparticles that achieved tumor‐selective drug accumulation and Anbazhagan et al.'s [25] RGD‐conjugated FA–paclitaxel polymer, which overcame chemoresistance in KB‐CH(R)‐8‐5 cells by modulating caspase‐3/9, p53, and Bax/Bcl‐2 balance. Collectively, these studies underscore how nanoencapsulation amplifies FA's therapeutic potential through targeted delivery and multimechanistic action. A summary of nano‐based applications of FA on cancer treatment is presented in Table 3 and Figure 6.

**Table 3:** Nano‐based application of ferulic acid (FA) in cancer treatment.

Formulation name	Study models	Finding a mechanism	Reference
FA encapsulated (250 ppm)	MCF‐7, 4T1	↓Viability ↑Apoptosis	[61]
FA–polyethylene oxide nanofibers	MCF‐7	↑Apoptosis	[62]
FA lipid nanocarriers (NCs)	HCT116, Caco‐2	↓Cyclin D1, IGF‐II, VEGF, ↑BAX/Bcl‐2 ratio ↑Antioxidant, anti‐inflammatory effects	[57]
FA–SLNs (25 μg/mL)	HT‐29	↑Cytotoxicity ↑Apoptosis via cytochrome c release, caspase 9, p53	[63]
FA‐layered double hydroxide nanohybrids	HeLa	↓Proliferation	[64]
ZnONPs‐FAC	Huh‐7, HepG2	↑Apoptosis via ROS generation ↑DNA damage ↓DEN‐enhanced HCC	[65]
FA‐loaded NLCs (36 µM)	U87‐MG	↑TG2 localization ↑Apoptosis via PARP‐1, caspase‐3 cleavage	[66]
FA‐loaded NLCs	Glioblastoma	↑Apoptosis via caspase‐3 cleavage ↓Bcl‐2, ERK1/2, c‐Myc ↑PARP‐1 cleavage	[67]
c‐SLNs of FA and ASP	MIA PaCa‐2, Panc‐1	↑Apoptosis via p‐RB, p21, p‐ERK1/2 ↓PCNA, MKI67 ↓Tumor growth	[55]
PFA&DOX nanoparticles	Tumor models	↑Drug accumulation at the tumor site, ↑Chemotherapeutic efficacy	[67]
FA–polymer with PTX (RGD‐PAMAM‐FP)	KB‐CH(R)‐8‐5	↑Apoptosis via caspase‐3, caspase‐9, p53, Bax ↓Antiapoptotic factors	[25]

Abbreviations: BAX, Bcl‐2 associated X protein; Bcl‐2, B‐cell lymphoma 2; c‐Myc, cellular myelocytomatosis oncogene; c‐SLNs, coloaded solid lipid nanoparticles; DEN, diethyl nitrosamine; ERK1/2, extracellular signal‐regulated kinases 1 and 2; FA, ferulic acid; HCC, hepatocellular carcinoma; HeLa, Henrietta Lacks; HER2, human epidermal growth factor receptor 2; IGF‐II, insulin‐like growth factor II; MCF‐7, Michigan Cancer Foundation‐7; MKI67, marker of proliferation Ki‐67; NLCs, nanostructured lipid carriers; PAMAM, polyamidoamine; PARP‐1, poly (ADP‐ribose) polymerase 1; PCNA, proliferating cell nuclear antigen; p‐ERK, phosphorylated ERK; PTX, paclitaxel; RGD, arginine–glycine–aspartic acid (motif); ROS, reactive oxygen species; SLNs, solid lipid nanoparticles; TG2, transglutaminase 2; VEGF, vascular endothelial growth factor; ZnONPs‐FAC, zinc oxide‐FA conjugates.

### 4.2 Synergistic Application of FA on Cancer Treatment

FA shows real promise in cancer therapy through its synergy with other agents, including natural products, vitamins, and conventional chemotherapeutic drugs. This combination enhances efficacy, reduces drug resistance, and reduces side effects such as ototoxicity, making FA a compelling adjunctive option to improve cancer treatment outcomes.

#### 4.2.1 Synergistic Efficacy of FA with Other Natural Products

FA shows significant synergistic efficacy when coadministered with other bioactive compounds, making it a promising adjunct for integrative cancer therapy. Studies reveal its diverse mechanisms across cancer types: (1) Cell cycle and proliferation: FA conjugated with resveratrol upregulated tumor suppressor p15 (a CDK inhibitor), reducing proliferation in HCT116 colorectal and MCF‐7 breast cancer cells [68]. (2) Metabolic Modulation: The FA/*p*‐coumaric acid combination inhibits PKM2 expression, suppressing aerobic glycolysis in HCT116/HT‐29 cells and decreasing intestinal polyps in C57BL/6J mice [69]. (3) Drug Resistance Reversal: IN A375 melanoma, FA combination with 2‐methoxyestradiol reduced Hsp60/90 and increased nitric oxide, bypassing resistance while leaving Hsp70 unchanged [70]. (4) Radio‐sensitization: FA combined with 2‐deoxyglucose made radiation therapy more lethal to NCI‐H460 lung cancer cells through p53/p21/NF‐κB activation alongside Ataxia‐Telangiectasia Mutated (ATM) suppression [71]. Collectively, these studies highlight FA's potential to amplify the effects of chemotherapy, radiotherapy, and targeted therapy via diverse molecular mechanisms.

#### 4.2.2 Synergistic Efficacy of FA With Vitamins

FA, in combination with vitamin E derivatives, shows powerful synergistic anticancer effects, especially with δ‐tocotrienol (δ‐T3), a vitamin E isoform known for its well‐established anti‐inflammatory, antioxidant, and antitumor activity. The combination of δ‐T3 and FA profoundly reduced telomerase activity and suppressed human telomerase reverse transcriptase expression in DLD‐1 colorectal adenocarcinoma cells, an effect not seen with FA treatment alone [72]. Eitsuka et al. [73] further showed that this combination triggers G1 cell cycle arrest in PANC via p21 upregulation, successfully reducing proliferation, while FA alone showed no significant activity. These findings underscore FA's role as a synergistic partner, boosting δ‐T3's therapeutic activity across diverse cancer types. On top of that, FA combined with unsaturated tocotrienols (TTs) demonstrated antiproliferative and proapoptotic effects, as well as the ability to reduce metastatic and angiogenic properties in various cancer cell types.

#### 4.2.3 Synergistic Efficacy of FA With Radiotherapy

Radiotherapy efficacy can be substantially enhanced by combining it with natural radiosensitizers, such as FA, a phenolic compound with prooxidant properties in cancer cells. Studies demonstrate FA's ability to potentiate radiation effects through multiple mechanisms. Karthikeyan et al. [74] showed that FA pretreatment amplifies radiotherapy‐induced cytotoxicity in cervical cancer (HeLa and ME‐180 cells) by (1) elevating lipid peroxidation and ROS generation, (2) causing oxidative DNA damage, and (3) inducing characteristic apoptotic morphology. Complementing these findings, Das et al. [75] reported that FA increased radiosensitivity in lung (A549) and liver (HepG2) carcinomas by (1) Inhibiting Akt/p38 MAPK survival signaling, (2) activating mitochondrial apoptosis (caspase‐3, nuclear fragmentation), and (3) arresting cell cycle progression. These dual studies position FA as a promising phytochemical radiosensitizer that operates through both oxidative stress potentiation and pathway‐specific modulation.

#### 4.2.4 Synergistic Efficacy of FA With Conventional Chemotherapies

FA acts as a potent chemosensitizer when co‐administered with standard chemotherapy agents, demonstrating enhanced anticancer effects while reducing treatment toxicity. Research reveals FA's unique synergy with gemcitabine, a frontline nucleoside analog used to treat pancreatic, lung, and breast cancers. Dodurga et al. [76] demonstrated that FA–gemcitabine combination therapy significantly amplified apoptotic responses and altered gene expression profiles in PC‐3 prostate cancer cells compared with gemcitabine monotherapy. FA enhances the efficacy of multiple chemotherapeutic agents through distinct mechanisms. In KB ChR8‐5 cells, FA potentiates doxorubicin and vincristine by promoting γ‐H2AX‐mediated DNA damage and modulating PI3K/Akt signaling [77]. For paclitaxel (PTX), FA‐loaded PAMAM dendrimers synergistically improve anticancer activity by upregulating caspase‐3/9, p53, and Bax while suppressing antiapoptotic factors [25].

In the HCT116 cell line, FA overcomes oxaliplatin resistance by targeting NEU3‐mediated glycosphingolipid metabolism and reducing p‐gp12 efflux [25]. FA shows synergistic effects when paired with diverse chemotherapeutic agents via various mechanisms. FA combined with the PARP inhibitor ABT‐888 (veliparib) enhanced DNA damage in breast cancer cells by impairing RAD51‐mediated repair and enhancing γ‐H2AX foci [78]. The FA–5FU combination therapy enhanced p53‐mediated apoptosis and reduced normal cell toxicity in colon cancer cell models [79]. Additionally, in triple‐negative breast cancer (MDA‐MB‐231 cells), FA (10 μM) boosted epirubicin (1 μM) effects through three mechanisms, including upregulation of caspase‐3, modulation of the Bcl/Bax balance, and induction of ER stress markers (protein disulfide isomerase, inositol‐requiring enzyme 1 alpha, and phosphoenolpyruvate carboxykinase) [80]. These findings demonstrate FA's diverse role as a chemosensitizer and chemoprotective adjuvant.

#### 4.2.5 Synergistic Efficacy of FA With Conventional Nonchemotherapy Drugs

**Figure 7:**
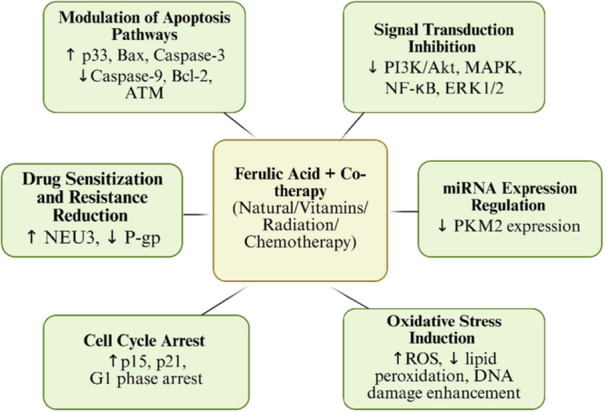
Synergistic application of ferulic acid (FA) on cancer treatment with other treatment strategies. Overview of the synergistic mechanisms of FA combined with co‐therapies, showing its roles in apoptosis, oxidative stress, cell cycle arrest, drug resistance reduction, signal inhibition, and miRNA regulation in integrative cancer therapy. ATM, Ataxia‐Telangiectasia Mutated; BAX, Bcl‐2 associated X protein; Bcl‐2, B‐cell lymphoma 2; ERK1/2, extracellular signal‐regulated kinases 1 and 2; miRNA, microRNA; NEU3, neuraminidase 3; NF‐κB, nuclear factor kappa‐B; P‐gp, *P*‐glycoprotein; PI3K, phosphoinositide 3‐kinase; PKM2, pyruvate kinase M2; ROS, reactive oxygen species. Created in BioRender. Sohel, M. (2026) https://BioRender.com/8prxhnf.

While aspirin (acetylsalicylic acid) is clinically employed for its analgesic, antipyretic, anti‐inflammatory, and antithrombotic properties in cardiovascular prevention, its combination with FA demonstrates novel anticancer potential. Thakkar et al. [55] developed c‐SLNs containing FA and aspirin that significantly enhanced antitumor efficacy through (1) apoptosis induction via p‐RB/p21/p‐ERK1/2 pathway activation, (2) cell cycle disruption, (3) dose‐sparing effects through improved cellular uptake, and (4) suppression of proliferation markers (PCNA and MKI67). These findings reveal a promising repurpose of aspirin‐FA combinations in oncology (see Table 4 and Figure 7 for comprehensive synergy data).

**Table 4:** Synergistic application of ferulic acid (FA) on cancer treatment.

Cancer type	Combined agents	Cell line	Combined target	Reference
Colon cancer	FA + resveratrol (yes)	HCT116, MCF‐7	↑p15 mRNA ↓Cell proliferation	[68]
	FA + *p*‐coumaric acid (yes)	HCT116, HT‐29	↓PKM2 ↓Aerobic glycolysis ↓Tumor polyps	[69]
Melanoma	FA + 2‐methoxyestradiol (yes)	A375	↓Hsp60 ↓Hsp90 ↑Nitric oxide ↓Drug resistance	[70]
Lung cancer	FA + 2DG + irradiation (yes)	NCI‐H460	↑Apoptosis ↑p53 ↑p21 ↑NF‐κB ↑Bax ↑Caspase‐3 ↓ATM gene expression	[71]
Colorectal cancer	FA + δ‐tocotrienol (yes)	DLD‐1	↓Telomerase activity, ↓hTERT expression	[73]
Pancreatic cancer	FA + δ‐tocotrienol (yes)	PANC	↑p21	[73]
Cervical cancer	FA + radiotherapy (yes)	HeLa, ME‐180	↑Lipid peroxidation ↑ROS levels ↑Oxidative DNA damage ↑Apoptosis	[74]
Lung cancer	FA + radiotherapy (yes)	A549	↑Akt/P38 MAPK modulation ↑Caspase‐3 ↑Nuclear fragmentation ↓Cell cycle progression	[75]
Liver cancer	FA + radiotherapy (yes)	HepG2	↑Akt/P38 MAPK modulation ↑Caspase‐3 ↑Nuclear fragmentation ↓Cell cycle progression	[75]
Prostate cancer	FA + gemcitabine (yes)	PC‐3	↑Apoptosis ↓Metastasis ↓Gene expression	[76]
Breast cancer	FA + doxorubicin + vincristine (yes)	KB ChR8‐5	↑γ‐H2AX foci formation ↑Apoptosis ↑PI3K/Akt modulation	[77]
Leukemia	FA + doxorubicin + vincristine (yes)	KB ChR8‐5	↑γ‐H2AX foci formation ↑Apoptosis ↑PI3K/Akt modulation	[77]
Lymphoma	FA + doxorubicin + vincristine (yes)	KB ChR8‐5	↑γ‐H2AX foci formation ↑Apoptosis ↑PI3K/Akt modulation	[77]
Ovarian cancer	FA + paclitaxel (yes)	PAMAM dendrimer	↑Caspase‐3 ↑Caspase‐9 ↑p53 ↑Bax ↓Antiapoptotic factors	[25]
Breast cancer	FA + paclitaxel (yes)	PAMAM dendrimer	↑Caspase‐3 ↑Caspase‐9 ↑p53 ↑Bax ↓Antiapoptotic factors	[25]
Lung cancer	FA + paclitaxel (yes)	PAMAM dendrimer	↑Caspase‐3 ↑Caspase‐9 ↑p53 ↑Bax ↓Antiapoptotic factors	[25]
Pancreatic cancer	FA + paclitaxel (yes)	PAMAM dendrimer	↑Caspase‐3 ↑Caspase‐9 ↑p53 ↑Bax	[25]
Colorectal cancer	FA + oxaliplatin (yes)	HCT116	↓NEU3 activity ↓Gb3 ↓P‐gp12	[25]
Breast cancer	FA + veliparib (yes)	Breast cancer cells	↓Recombination repair ↓RAD51 foci formation ↑γ‐H2AX accumulation	[78]
Colorectal cancer	FA + 5‐fluorouracil (5‐FU) (yes)	In silico and Wistar‐albino rats	↑p53 ↑DNA repair ↑Apoptosis ↓Cytotoxicity	[12]
Breast cancer	FA + epirubicin (yes)	MDA‐MB‐231 cells	↑Bax ↑Caspase‐3 ↑PDI ↑IRE1α ↑PEPK ↓Bcl‐2	[79]
Pancreatic cancer	FA + aspirin (yes)	c‐SLNs model	↑Apoptosis ↑p‐RB ↑p21 ↑p‐ERK1/2 ↓PCNA ↓MKI67 ↓Tumor growth	[55]

Abbreviations: 2DG, 2‐deoxyglucose; Akt, protein kinase B; ATM, Ataxia‐Telangiectasia Mutated; BAX, Bcl‐2 associated X protein; Bcl‐2, B‐cell lymphoma 2; c‐SLNs, coloaded solid lipid nanoparticles; ERK1/2, extracellular signal‐regulated kinases 1 and 2; Gb3, globotriaosylceramide; HeLa, Henrietta Lacks; Hsp, heat shock protein; hTERT, human telomerase reverse transcriptase; IRE1α, inositol‐requiring enzyme 1 alpha; MAPK, mitogen‐activated protein kinase; MB, membrane bound; MDA, malondialdehyde; MKI67, marker of proliferation Ki‐67; mRNA, messenger RNA; NEU3, neuraminidase 3; NF‐κB, nuclear factor kappa‐B; PAMAM, polyamidoamine; PANC, pancreatic cancer; PCNA, proliferating cell nuclear antigen; PDI, protein disulfide isomerase; PEPK, phosphoenolpyruvate carboxykinase; p‐ERK, phosphorylated ERK; P‐gp12, *P*‐glycoprotein 12; PI3K, phosphoinositide 3‐kinase; PKM2, pyruvate kinase M2; RAD51, RAD51 recombinase; ROS, reactive oxygen species; γ‐H2AX, phosphorylated H2A.X (histone H2A variant X).

## 5 The Mechanisms of FA in Alleviating Multidrug Resistance (MDR) and Reversing the Active Form in Cancer

Like many other natural compounds, FA can help fight MDR in various cancers by regulating drug efflux pumps, boosting drug uptake, and counteracting key MDR mechanisms, thereby improving chemotherapy efficacy.

### 5.1 Focusing on the MDR Protein ABCB1/*P*‐Glycoprotein (P‐gp)

The MDR protein ABCB1, also known as P‐gp, is located on the plasma membrane and plays a key role in chemoresistance by actively pumping anticancer drugs from tumor cells through ATP‐dependent efflux. Recent studies show that FA can counteract this resistance mechanism through several molecular pathways. Muthusamy et al. [77] reported that FA enhances the effectiveness of doxorubicin and vincristine in P‐gp‐overexpressing KB ChR8‐5 cells by inhibiting nuclear factor kappa‐B (NF‐κB) nuclear translocation via the PI3K/Akt pathway, which in turn downregulates P‐gp expression and restores drug sensitivity. Similarly, Anbazhagan et al. [25] found that FA increases paclitaxel accumulation in resistant KB CH‐R 8‐5 cells by modulating P‐gp activity, and activating caspase‐3/9, p53, and Bax, while suppressing antiapoptotic factors. Supporting these findings, Muthusamy et al. [81] further showed that FA‐mediated suppression of ABCB1 expression restores paclitaxel‐induced cycle arrest and apoptosis in resistant cell lines. Collectively, these studies highlight FA as a promising chemosensitizer that can overcome P‐gp‐mediated MDR through multiple interacting molecular mechanisms.

### 5.2 Sensitizing Chemo and Radiotherapies

FA demonstrates potent radiosensitizing and chemosensitizing properties that enhance conventional cancer therapies through multiple mechanisms. As a radiosensitizer, FA potentiates radiation effects by (1) increasing oxidative stress through elevated lipid peroxidation and ROS generation, (2) inducing DNA damage, and (3) promoting apoptotic cell death—as demonstrated in cervical (HeLa and ME‐180) and hepatopulmonary (A549 and HepG2) cancer models [74, 75]. These effects are mediated by modulation of key pathways, including Akt/p38 MAPK signaling and mitochondrial apoptosis. As a chemosensitizer, FA overcomes MDR by enhancing DNA damage markers (γ‐H2AX foci), activating apoptotic pathways, and regulating PI3K/Akt signaling, thereby significantly improving the cytotoxicity of doxorubicin and vincristine in resistant ChR8‐5 cells [77]. Importantly, FA achieves these therapeutic enhancements while potentially reducing treatment‐related adverse effects, making it an attractive adjunct in combinatorial oncology regimens. Similarly, FA effectively increased the efficacies of some existing drugs, like paclitaxel [25], oxaliplatin [25], ABT‐888 [78], cisplatin [82], 5‐fluorouracil [12], and epirubicin [80], indicating the increasing activity of the drugs by FA.

### 5.3 Conversion of EMT

The EMT endows cancer cells with more aggressive characteristics, such as enhanced migration and invasiveness. This process involves the downregulation of epithelial markers (e.g., E‐cadherin), upregulation of mesenchymal proteins (e.g., vimentin), and activation of EMT‐related transcription factors, like Snail and Slug. Together, these changes contribute to therapeutic resistance by decreasing drug uptake, enhancing survival pathways, and enabling cells to evade apoptosis. Notably, FA has shown strong antimetastatic activity in triple‐negative breast cancer (MDA‐MB‐231 cells) by reversing EMT. Specifically, FA (1) increases E‐cadherin (epithelial marker) expression, (2) decreases vimentin (mesenchymal marker) levels, and (3) suppresses EMT‐inducing transcription factors (Snail and Slug) [9]. These results suggest that FA may help restore epithelial phenotypes and reduce therapy resistance in highly invasive cancers.

### 5.4 Apoptosis Regulation

The dysregulation of apoptotic mechanisms confers a survival benefit to cancer cells, enabling them to develop MDR—a hallmark of aggressive tumors that become refractory to multiple structurally and functionally diverse chemotherapeutic agents [83]. FA counteracts this resistance by upregulating key cell cycle regulators (p53 and p21) while modulating the expression of apoptotic mediators, including procaspase‐3, procaspase‐8, procaspase‐9, PARP, and the Bcl‐2/Bax ratio. Through these coordinated actions on both cell cycle and apoptotic pathways, FA effectively restores cancer cell sensitivity to chemotherapeutic drugs [15].

### 5.5 Targeting ROS Production in Cancer Cells

While elevated ROS can be cytotoxic to cancer cells, tumors often develop chemoresistance by upregulating antioxidant defenses to neutralize drug‐induced oxidative stress [84]. FA counteracts this adaptation through multiple mechanisms: Cao et al. [26] demonstrated that FA treatment in EC‐1 and TE‐4 esophageal cancer cells increases ROS while reducing antioxidant capacity (SOD and GSH depletion), while simultaneously inducing lactate dehydrogenase release and caspase‐3 activation to promote cell death. Similarly, Preedia Babu et al. [65] showed that zinc oxide‐FA conjugates (ZnONPs‐FAC) induce ROS‐mediated DNA damage and apoptosis in HCC (Huh‐7 and HepG2) cells and suppress DEN‐induced carcinogenesis. In radiotherapy contexts, Karthikeyan et al. [74] reported that FA pretreatment enhances radiation effects by amplifying lipid peroxidation, ROS accumulation, DNA damage, and apoptotic morphology in cervical cancer cells (HeLa and ME‐180).

### 5.6 Hypoxia Regulation

Hypoxia‐inducible factors (HIFs) serve as critical mediators that enable cancer cell survival under hypoxic conditions, promoting chemoresistance through multiple synergistic pathways [85]. At 50 mg/kg, FA exhibits significant antitumor activity against ESC by specifically suppressing HIF‐1α signaling. This suppression in turn reshapes several downstream pathways, including (1) activation of the Nrf2/HO‐1 oxidative stress response, (2) inhibition of proliferative regulators, such as c‐Myc and cyclin D1, and (3) downregulation of key metabolic and survival mediators, mTOR and STAT3 [46].

### 5.7 MicroRNAs (miRNAs) Regulation

The dysregulation of noncoding RNAs, particularly miRNAs and lncRNAs, plays a central role in establishing MDR in cancer cells. FA‐loaded TPGS micelles counteract this by targeting the miRNA‐221/TP53INP1 axis, providing a dual mechanism of action: they suppress tumor‐promoting miRNA‐221 while simultaneously boosting autophagy‐related pathways via TP53INP1c [63].

### 5.8 Targeting the β‐Catenin Pathway

β‐Catenin, a central mediator of Wnt signaling, promotes chemoresistance in cancer cells by enhancing drug efflux. Qifa et al. [32] showed that isoferulic acid (IFA) effectively overcomes this resistance in renal carcinoma (A‐498 cells) through two coordinated mechanisms: (1) downregulation of β‐catenin expression and (2) activation of apoptosis via increased cleavage of PARP and caspase‐3.

### 5.9 Suppression of Oncogene Activation

**Figure 8:**
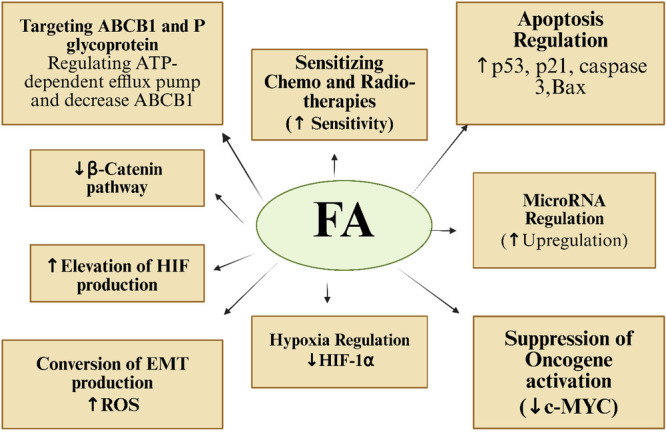
Schematic representation of the molecular mechanisms of FA in cancer therapy. FA can modulate multiple pathways, including apoptosis regulation, hypoxia modulation, EMT conversion, β‐catenin suppression, HIF elevation, microRNA regulation, oncogene inhibition, and enhanced sensitivity to chemo‐ and radiotherapy. ABCB1, ATP‐binding cassette subfamily B member 1; ATP, adenosine triphosphate; BAX, Bcl‐2 associated X protein; c‐Myc, cellular myelocytomatosis oncogene; EMT, epithelial–mesenchymal transition; FA, ferulic acid; HIF‐1α, hypoxia‐inducible factor‐1 alpha; ROS, reactive oxygen species. Created in BioRender. Sohel, M. (2026) https://BioRender.com/8prxhnf.

Oncogene activation in cancer drives therapeutic resistance through multiple mechanisms, including survival pathway activation, drug target modification, enhanced DNA repair, upregulation of efflux pumps, and adaptive evasion strategies [86]. FA counteracts these resistance mechanisms through distinct pathways: (1) In glioblastoma (U‐87 MG cells), FA triggers ROS‐mediated ERK1/2 activation, creating a proapoptotic environment characterized by reduced Bcl‐2 and ERK1/2 expression, alongside c‐Myc suppression [30]. (2) In renal carcinoma (A‐498 cells), IFA promotes apoptosis through c‐Myc downregulation and subsequent activation of cleaved PARP and caspase‐3 [32]. Together, these findings demonstrate FA's multimodal action against oncogene‐driven resistance (summarized in Table 5 and Figure 8), positioning it as both a potent chemosensitizer and apoptosis inducer across diverse cancer types.

**Table 5:** The mechanisms of ferulic acid (FA) in alleviating multidrug resistance and reverse active form in cancer.

Mechanism	Effect of FA	Key findings	Reference
Regulating drug efflux pumps	↓P‐gp levels ↑Drug uptake	↓NF‐κB ↓P‐gp ↑Apoptosis	[77]
Enhancing drug sensitivity	↑Chemotherapy efficacy	↑Drug availability ↑Apoptosis ↑Caspase‐3 ↑Caspase‐9, p53 ↑Bax	[25]
Sensitizing chemo and radiotherapy	↑Chemotherapy outcomes ↑Radiotherapy outcomes	↑ROS ↑Lipid peroxidation ↑DNA damage ↑Sensitizing cancer cells to treatment	[74, 75]
Conversion of EMT	↓EMT ↓Metastatic potential	↓E‐cadherin ↓Vimentin ↓Snail ↓Slug ↓EMT	[9]
Apoptosis regulation	↑Apoptosis sensitivity	↑p53 ↑p21 ↑Caspases ↓Antiapoptotic signals	[15]
Targeting ROS production	↑ROS ↑DNA damage ↑Apoptosis	↑ROS ↓Antioxidant defenses ↑Caspase‐3	[26, 65, 74]
Hypoxia regulation	↓HIF‐1α	↑Nrf2 ↑HO‐1 ↑c‐Myc ↑Cyclin D1 ↑mTOR ↑STAT3 ↓Hypoxia‐induced resistance	[85]
MicroRNA regulation	↓miRNA ↓lncRNA	↓miRNA‐221/TP53INP1 ↑Apoptosis ↑Autophagy	[63]
β‐Catenin pathway targeting	↓β‐Catenin ↑Apoptosis	↑PARP ↑Caspase‐3	[32]
Suppression of oncogene activation	↓Oncogene‐driven drug resistance	↓Bcl‐2 ↓ERK1/2 expression ↑c‐Myc ↑Apoptosis	[30]

Abbreviations: BAX, Bcl‐2 associated X protein; Bcl‐2, B‐cell lymphoma 2; c‐Myc, cellular myelocytomatosis oncogene; EMT, epithelial–mesenchymal transition; ERK1/2, extracellular signal‐regulated kinases 1 and 2; HIF‐1α, hypoxia‐inducible factor‐1 alpha; HO‐1, heme oxygenase‐1; lncRNA, long noncoding RNA; miRNA, microRNA; mTOR, mammalian target of rapamycin; NF‐κB, nuclear factor kappa B; Nrf2, nuclear factor erythroid 2‐related factor 2; PARP, poly (ADP‐ribose) polymerase; P‐gp, *P*‐glycoprotein; ROS, reactive oxygen species; STAT3, signal transducer and activator of transcription 3.

## 6 Limitations and Future Perspectives

Despite our review indicating that FA has potential anticancer activity, to better determine its therapeutic advantages, some issues still need to be addressed. For example, a therapeutic dose, because the current literature shows that FA is effective at various doses across several cancer types. This kind of inconsistency really demands more study to determine a definitive dose for each cancer type. Additionally, although FA is effective in the majority of in vitro models, a few preclinical studies also require robust in vivo models to validate clinical efficacy and safety. Some studies have shown that FA has lower bioavailability and solubility, which may make it less therapeutically useful. Another potential pitfall for FA is the lack of established treatment protocols, which hinders clinical practice. To address these issues, researchers need to focus on nanoformulation of FA, which will improve solubility and efficacy while minimizing side effects. FA's synergistic effects with other chemotherapeutic agents, natural compounds, and radiotherapy also offer a promising avenue for combination therapies, which could improve overall treatment efficacy. To facilitate its clinical use, more extensive human clinical trials are needed to establish optimal dosing guidelines, safety profiles, and therapeutic effectiveness. FA can be used in personalized medicine, which is effective for specific genetic mutations.

## 7 Conclusion

According to this literature review, FA has the potential to work against several types of human cancer. FA regulates oncogenic signaling pathways related to cell proliferation (e.g., PI3K/AKT and MAPK), apoptosis (Bcl‐2/Bax and caspase cascades), metastasis (MMP inhibition), and chemoresistance (P‐gp downregulation). This mechanism supports FA's pharmacological properties, making it a candidate for the development of cancer drugs. The most unique feature is that FA selectively shows its toxicity toward cancer cells rather than healthy cells. FA is active synergistically either with standard drugs or in combination with other phytochemicals. In addition, nanoformulation of FA can improve its bioavailability, solubility, stability, and controlled release, thereby increasing its therapeutic efficacy. Despite these promising findings, more clinical research is required to validate its toxicity and clinical effectiveness through a comprehensive clinical trial.

## Author Contributions

**Sanzida Khatun:** conceptualization, writing – original draft. **Md Sohel:** conceptualization, writing – original draft. **Zitu Barman:** writing – original draft. **Umme Salma:** writing – original draft. **Lubatul Arbia:** writing – original draft. **Rifat Sarkar:** writing – original draft. **Jasmin Akter Jame:** writing – original draft. **Badhon Rani Dey:** writing – original draft. **Sultana Parvin:** writing – original draft. **Md. Shah Poran Shuvo:** writing – original draft. **Md. Shahidul Islam:** writing – original draft. **Tania Mannan:** writing – review and editing. **Snygdha Rani Das:** writing – original draft, writing – review and editing. **Md. Mahmudul Hasan:** writing – original draft, writing – review and editing.

## Funding

The authors received no specific funding for this work.

## Ethics Statement

The authors have nothing to report.

## Consent

The authors have nothing to report.

## Conflicts of Interest

The authors declare no conflicts of interest.

## Data Availability

Data are included in the article/referenced in the article.
